# Supplementary material for: Forest growth responds more to air pollution than soil acidification
Source: PLoS One. 2023 Mar 8;18(3):e0256976. doi: 10.1371/journal.pone.0256976 (PMC9994739; doi:10.1371/journal.pone.0256976)
Supplement: S1 Annex — (DOCX) [file pone.0256976.s001.docx]

S1 Annex. MAGIC modelling

**S1.1. MAGIC calibration**

Estimated and measured total deposition constrains the model simulation. To predict theoretical soil chemistry (until the year 2050), all model parameters were fixed to the year 2018 (Table S1.1), as well as atmospheric deposition fluxes. Sulfur deposition (Fig. 5) is the most important driving factor for soils and water dynamics.

The MAGIC model does not explicitly model treatments such as the liming. To account for and predict the effects of the liming (Scenarios Liming in all figures), Ca, respectively Mg inputs were entered as external inputs to the model (source approach). Limestone/dolomitic limestone dissolution was estimated as:

$$F_{Cai,Mgi}=T{OT}_{Ca,Mg}*{exp}^{-k*}$$

where F_Cai,Mgi_ is a fraction of Ca or Mg released from the solid phase of limestone. TOT_Ca,Mg_ is the amount of applied limestone, parameter k represents the dissolution rate of Ca^2+^ and Mg^2+^ from the limestone/dolomitic limestone, respectively. The k parameter was set equal to 0.02 for Ca and 0.04 for Mg. It was derived from the MAGIC calibration procedure of the Načetín limed site where soil water data were available for 2007-2018. Dissolution dynamics at particular sites differ based on dose of limestone and time of application (Fig. S1.1).

**Figure S1.1.1. Annual fluxes of Ca^2+^ and Mg^2+^ released from limestone/dolomitic limestone at Načetín, Kovářská and Fláje (1980 – 2050).**

The calibration started from an assumed initial steady-state condition (here the year 1850). The model simulated pools of base cations, C and N in the soil, and concentrations of strong acid anions (SAA = SO_4_+Cl+NO_3_), base cations (SBC = Ca+Mg+Na+K), organic acids, acid neutralising capacity (ANC = SAA-SBC), Al and pH in soil and soil-water for each year through the end of the simulation period (here 2018). A trial-and-error procedure was used to obtain the best match between simulated and observed (measured) soil base cations, Al, pH, SAA and SBC in the calibration year 2018. MAGIC was calibrated at Načetín to the observed mean annual concentrations and fluxes of SO_4_, Cl, NO_3_, NH_4_, Ca, Mg, K, Na, Al and protons (pH) in soil water, and to the observed C/N ratio and base saturation (BS) in the soil.

**Table S1.1.1. Fixed and calibrated parameters used for the MAGIC simulations.**

| **Fixed parameters** | **Units** | Načetín | Kovářská | Fláje |
| --- | --- | --- | --- | --- |
| Average soil depth | m | 0.4 | 0.4 | 0.4 |
| Bulk density | kg·m^-3^ | 548 | 608 | 188 |
| Cation exchange capacity (CEC) | meq·kg^-1^ | 51 | 50 | 92 |
| Precipitation | mm | 1050 | 1050 | 1050 |
| Runoff | mm | 407 | 407 | 407 |
| Organic acids | mmol·m^-3^ | 65 | 65 | 100 |
| pK_1_ (organic acids) | -log | 3.5 | 3.5 | 3.5 |
| pK_2_ (organic acids) | -log | 4.4 | 4.4 | 4.4 |
| pK_3_ (organic acids) | -log | 5.5 | 5.5 | 5.5 |
| Net base cations(Ca+Mg+Na+K) uptake by vegetation | meq·m^-3^ | 12.2 | 12.2 | 12.2 |
| Soil base saturation (2018) | % | 5.8 | 38 | 64 |
| Soil C/N (2018) | mol·mol^-1^ | 23 | 23 | 23 |
| **Calibrated parameters** |  |  |  |  |
| Solubility Al(OH)_3_ | log | 9.2 | 9.2 | 9.1 |
| Base cations (Ca+Mg+Na+K) weathering | meq·m^-2^·yr^-1^ | 28.5 | 28.5 | 62 |
| Initial (1850) base saturation | % | 18.7 | 18.7 | 46 |
| SO_4_ adsorption half saturation | meq·m^-3^ | 95 | 100 | 100 |
| SO_4_ max. adsorption capacity | meq·kg^-1^ | 50 | 50 | 50 |
|  | | |  |  |
|  | | |  |  |

**S1.2. Modelling results**

**Načetín**

Detailed data on atmospheric deposition, soil water and soil chemistry were at Načetín control since 1994, and they were used for model calibration (Fig. S1.2). Peak concentrations of the sum of strong anions (SSA = SO_4_+NO_3_+Cl) in soil water were observed during the 1980s. Since then, a steady decrease in SAA was observed. The trend in measured data was closely simulated by the MAGIC model (Fig S1.2). Soil water concentration of the SBC followed the SAA with a peak during the acidification driven dominantly by SO_4_. Long-term simulated annual pH and ANC fitted the observed data (1994-2018). The increase of soil water acidity, following soil acidification by S deposition, peaked in the 1980s with soil water pH ca. 4.3 (Fig. 8), ANC ≈ -1400 µeq·l^-1^. Concentrations of inorganic Al reached levels ≈ 1200 µeq·l^-1^. The natural recovery phase after the year 1989 resulted in a rapid increase of ANC, a moderate increase of pH and a rapid decrease of Ali. Liming of the experimental subplot in 2017 did not affect SAA, but SBC and soil pH have increased substantially. ANC was affected less pronounced, and Ali declined by 1/3 compared to unlimed control (Fig S1.2). Overall, the simulated effects of the liming on soil chemistry agreed well with measured data. It shows the ability of the MAGIC model and the estimate of limestone dissolution to mimic the observed data and thus provide reasonable estimates of past and future soil chemistry.

**Figure S1.2.1. Modelled (1850-2050) and measured (1994-2018) soil water chemistry at Načetín (control and limed sites)**

**Kovářská**

Kovářská was geochemically very similar to Načetín (Tab. 1), thus estimated soil chemistry (Tab S1.1 and Fig. S1.3) resulted in very identical projections of soil water chemistry at the unlimed (control) scenario. Kovářská was limed earlier and repeatedly (cumulative dose of 6.5 t·ha^-1^ since 1981). It resulted in higher SBC, which peaked around 1990 at 600 µeq·l^-1^ , then decrease following the decline of SAA. ANC was modelled around 0 µeq·l^-1^ since 2020, and Ali declined to 20% of the estimated unlimed scenario (Fig. S1.3).

**Figure S1.2.2. Modelled soil water chemistry at Kovářská**

**Fláje**

Bedrock at Fláje provided more natural buffering of incoming acidity in comparison with other plots. Initial base saturation, as well as weathering of base cations (Tab. S1.1), were higher in comparison to gneiss underlined Načetín and Kovářská (Tab S1.1). Acid rain did not deplete base cations concentrations (Fig. S1.4) and liming since 1981 increased SBC higher than other sites (Fig. S1.2, S1.3). As a result, ANC reaches positive values +50 µeq·l^-1^ in the 2010s. Ali was an order of magnitude lower than the estimated control (no liming) scenario. Present acid-base chemistry at Fláje is significantly more base-rich that its preindustrial estimate (Fig. S1.4 and Fig. 8 in the main text).

**Figure S1.2.3 Modelled soil water chemistry at Fláje.**

**S1.3. Relationship between S deposition and TRW**

**Figure S1.3.1. Modelled (1850-1993) and measured (1994-2017) S deposition, measured TRW index during tree lifetime at Načetín, Kovářská and Fláje.**
