# Supplementary figures and images for: Forest growth responds more to air pollution than soil acidification
Source: PLoS One. 2023 Mar 8;18(3):e0256976. doi: 10.1371/journal.pone.0256976 (PMC9994739; doi:10.1371/journal.pone.0256976)

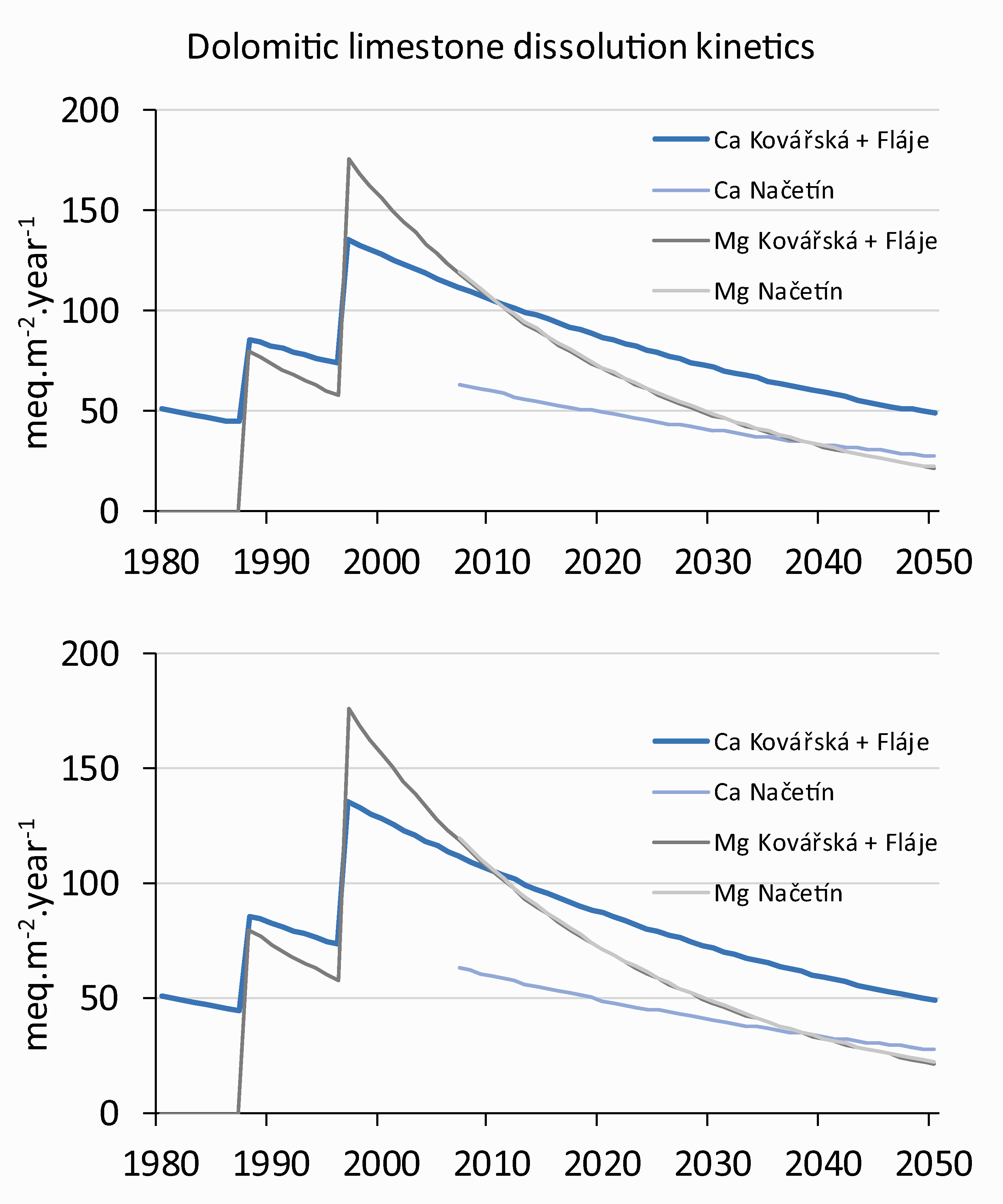

Supplement: S1 Fig — (TIF) [file pone.0256976.s003.tif]

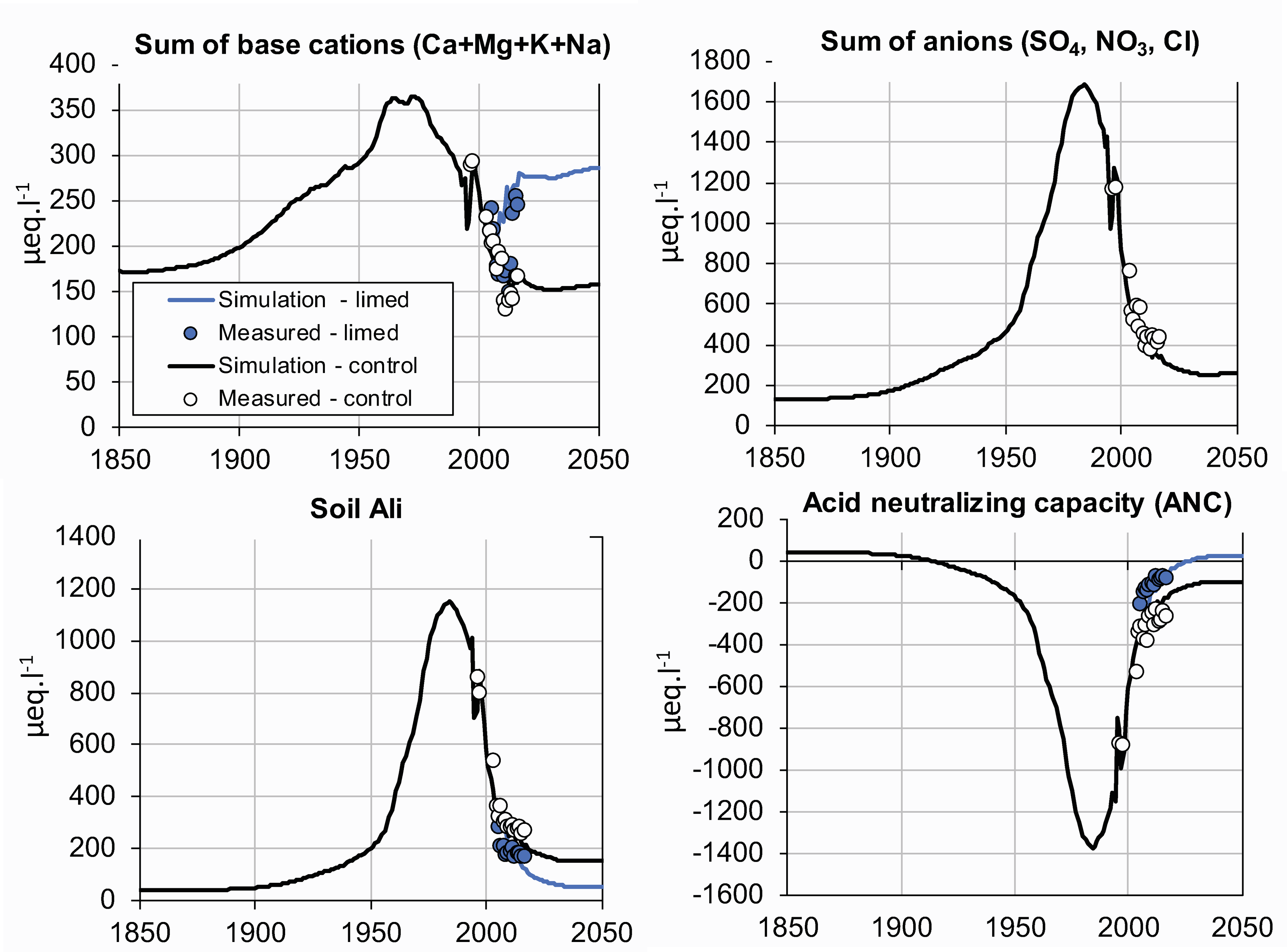

Supplement: S2 Fig — (TIF) [file pone.0256976.s004.tif]

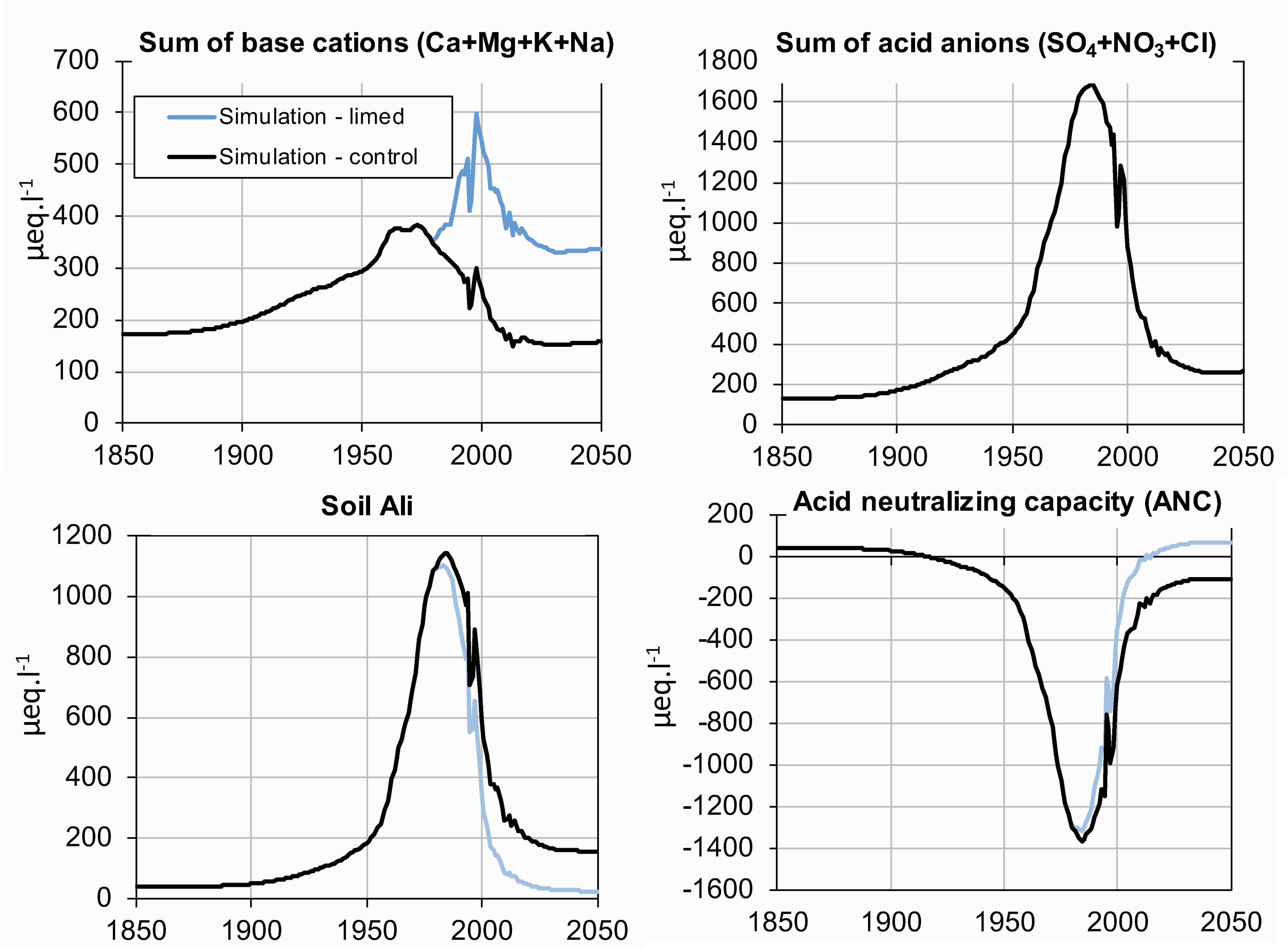

Supplement: S3 Fig — (TIF) [file pone.0256976.s005.tif]

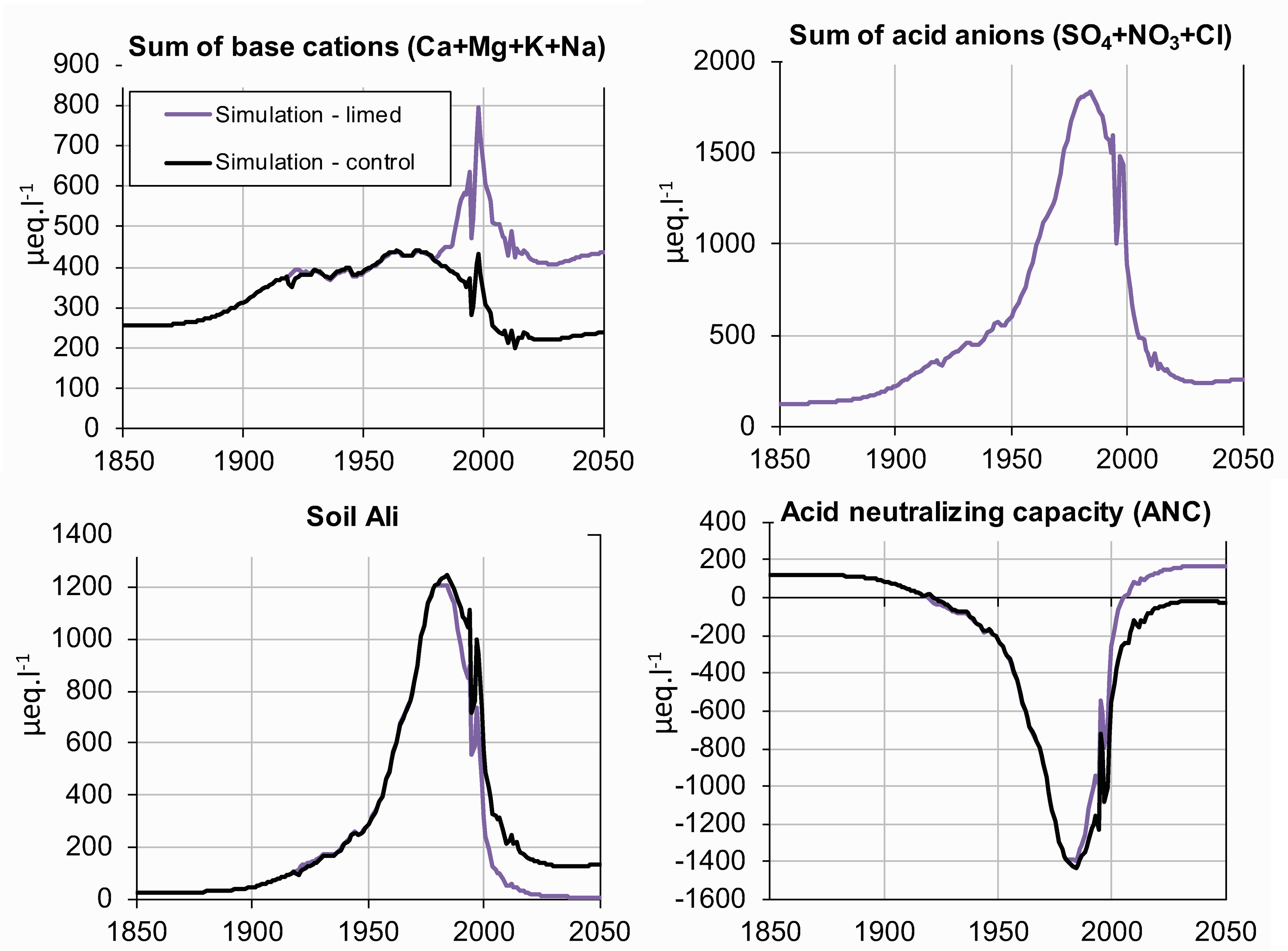

Supplement: S4 Fig — (TIF) [file pone.0256976.s006.tif]

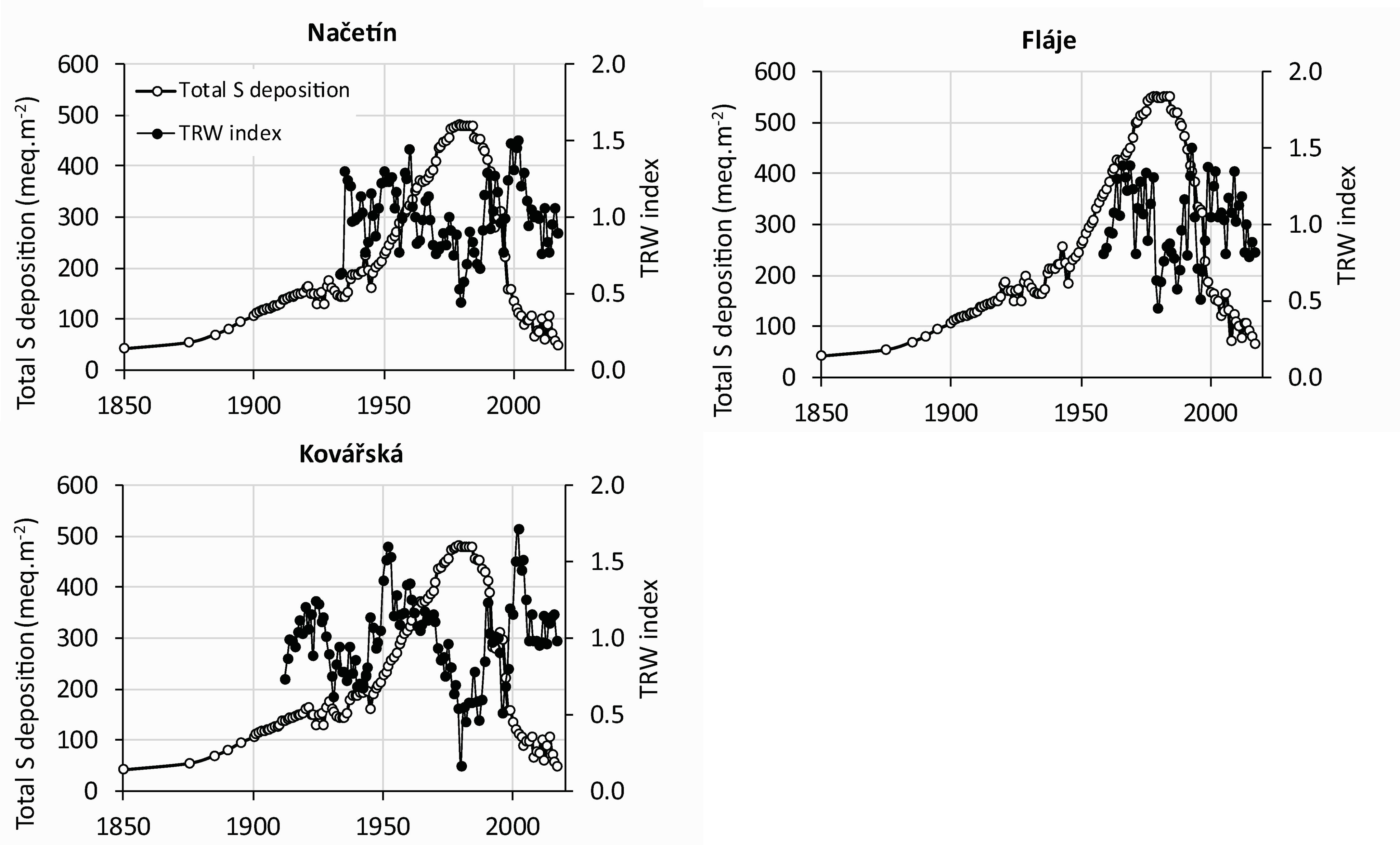

Supplement: S5 Fig — (TIF) [file pone.0256976.s007.tif]
